# Multiple central giant cell granuloma of the jaws: diagnostic signposts of Noonan syndrome and RASopathy

**DOI:** 10.1007/s10006-024-01209-2

**Published:** 2024-02-13

**Authors:** Reinhard E. Friedrich, Rico Rutkowski, Martin Gosau

**Affiliations:** Department of Oral and Craniomaxillofacial Surgery, Eppendorf University Hospital, University of Hamburg, 20246 Hamburg, Germany

**Keywords:** Noonan syndrome, Central giant cell granuloma, Jaw, RASopathy

## Abstract

Noonan syndrome (NS) is a phenotypically variable inherited multi-system disorder. Maxillofacial findings can be diagnostic, especially in the evaluation of discrete facial dysmorphia. Diagnostic landmark findings of therapeutic relevance for the jaws such as central giant cell granuloma (CGCG) are rare in NS. However, recent molecular genetic studies indicate that these rare, benign lesions are neoplasms and more common in specific syndromes grouped under the umbrella term RASopathies. A specialist surgical diagnosis can be helpful in identifying the underlying disease. This report outlines diagnosis and treatment of a case of CGCG for which jaw diagnosis became the key to identifying a syndromic disease.

## Introduction

Noonan syndrome (NS) is rare inherited multi-system disorder [1]. Approximately 50% of cases occur sporadically, meaning that the parents of affected children are not carriers of NS. NS is highly genetically heterogeneous. Approximately half the people with Noonan syndrome have mutations of the tyrosine-protein phosphatase non-receptor type 11 (PTPN11) gene, which is located on the long arm of chromosome 12 [2]. The gene encodes for an enzyme, Src homology region 2 domain–containing phosphatase-2 (SHP-2). SHP-2 is a protein tyrosine phosphatase involved in the signaling pathways of a variety of growth factors and cytokines [3]. The NS phenotype is multifaceted, and diagnosis can be difficult with inconspicuous findings. Therefore, the diagnosis is frequently delayed in oligosymptomatic cases [4]. However, the disease is relatively common with a presumed prevalence of 1:1000 to 1:2000 individuals [3, 4]. Seminal findings include combinations of cardiac alterations (e.g., congenital heart defects, cardiomyopathy), endocrine changes, and neoplasms already developing in childhood. NS patients are often of smaller body sizes than the average population [1]. The common facial dysmorphic characteristics, such as frontal bossing and hypertelorism, are not typical of a single condition. Rather, certain facial dysmorphias are frequently observed in individuals whose genetic modifications exert a persistent reinforcing effect on a signaling pathway regulated by the proto-oncogene Rat sarcoma (RAS) homolog. The mutations detectable in NS affect the RAS signaling pathway. For this reason, NS is classified in a group of heritable syndromes currently designated RASopathies [2, 5].

In NS, the skeleton may be generalized (short stature) or locally affected. Local skeletal influences of the syndrome, for example, on the facial skull, are high palate or microgenia. In rare cases, the jaws may develop central giant cell granulomas (CGCG) in association with NS. The jaw lesion is rare, as such not specific, and may arise in sporadic or syndromic patients. Syndromic CGCG is found clustered in RASopathies [6]. Apparently, CGCG is a neoplastic lesion caused by mutations in the RAS signaling pathway in both sporadic and syndromic cases [7]. Findings such as jaw swelling and tooth loosening may be early indications of CGCG [7}. The diagnosis of a CGCG and the suspicion of a syndromic background of the patient should be reasons for extended diagnostics [8].

The following report describes the diagnosis and treatment of a young patient with jaw lesions that were key to the identification of syndromic disease.

## Case report

### Medical history

The 15-year-old female patient was referred to the clinic for treatment of the maxillary lesion. Medical history concluded that the patient had developed left-side palate swelling some weeks ago and sought dental treatment. The patient reported that several teeth on the left side of the upper jaw had already come loose at this point. Suspecting an odontogenic abscess, tooth 26 was trepanned and a palatal soft tissue incision of the lesion was made, which was followed without draining of pus. Thereafter, the patient was referred to the clinic for further diagnosis and treatment. The mother’s interview revealed that medical examinations had been performed repeatedly and unsuccessfully because of the obvious physical developmental delay.

### Findings

On admission, the healthy, small patient (height: 154.6 cm, weight: 36.3 kg) showed pain-free habitual occlusion and slightly elongated first and second molars on the left side as a conspicuous feature, resulting in a right-sided open bite. On the left side of the hard palate, a prominent soft tissue tumor, characterized by pressure pain, had developed in the apical region of teeth 24 to 27. The mucosa showed no abnormalities except for the healing stitching incision. Teeth 25 and 26 did not respond to adequate cold stimuli.

In addition to the patient’s small stature, the delayed sexual development was conspicuous. The hormones LH, FSH, and estradiol were 0.41, 5.8, and 36 ng/l, respectively, within the normal range for the pubertal phase. There was a vitamin D deficiency (Vit. D3 = 15.4 µg/l). IGF-1 was measured in the pathologically low range (159 ng/ml, normal range: 190–429 ng/ml). Further findings were light curly hair, light-colored eyes, a high-arched palate, cubitus valgus, indicated pterygium, and focal skin pigmentation. The medial interorbital distance at the dacryon was 2.35 cm. A recent X-ray examination of the hand predicted a bone age of 12.5 years.

**Fig. 1:**
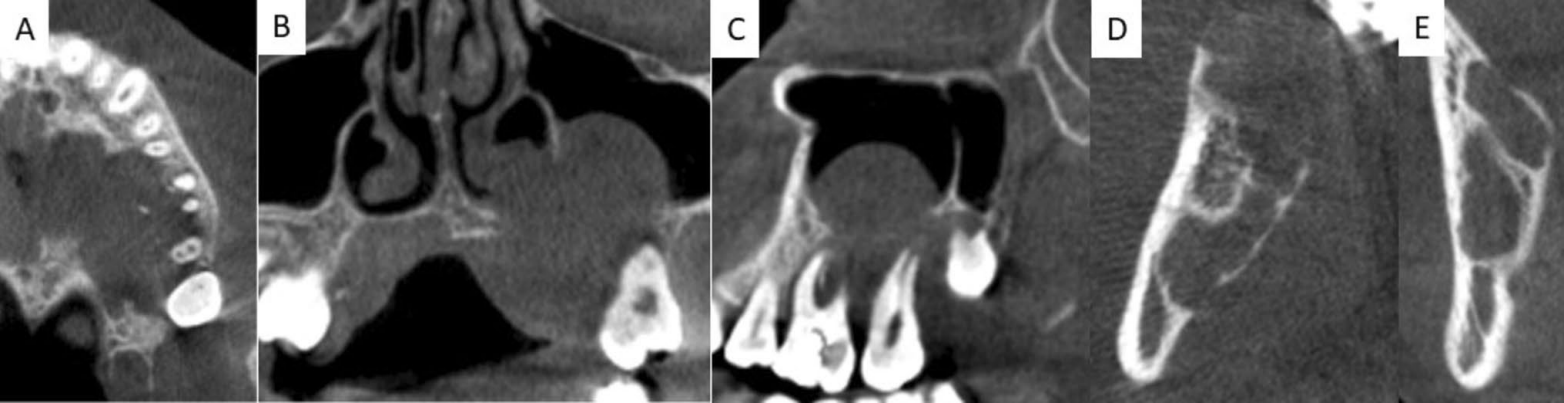
Radiological imaging of the jaw lesions of multilocular central giant cell granuloma of a Noonan syndrome patient in cone beam computed tomography (CBCT) prior to surgery ((**A**–**C**): maxilla (left side), (**D**, **E**): mandible (right side)). CBCT images show extensive osteolysis of the maxilla (**A**, **B**, **C**) and mandible (**C**, **D**) (section plane: (**A**): axial; (**B**) and (**E**): coronal; (**C**+**D**): sagittal)

**Fig. 2:**
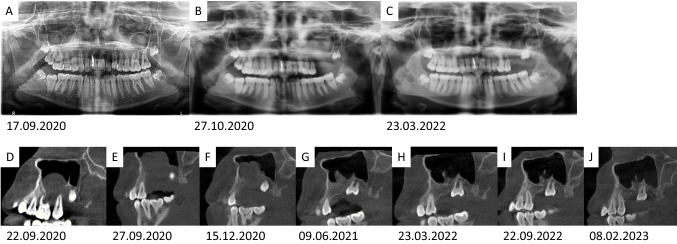
Follow-up of the maxillary and mandibular central giant cell granuloma (CGCG) in the panoramic view (**A**–**C**) and the cone beam computed tomography (**D**–**J**, sagittal projections). The subtotal re-ossification of the right mandibular angle can be seen on the panoramic views. An area around the nerve canal remains hypointense and with a garland-shaped outline. In the maxilla, (**D**) shows the initial findings, (**E**) the condition after the first resection (soft tissue swelling), followed by (**F**) the persistence of the mass in the maxillary sinus and the change in shape (recurrent CGCG). (**G**–**J**) illustrate maxillary bone formation over time and further development of the upper left wisdom tooth. The tooth shows a considerable physical mesial drift into the newly formed bone

On MRI, a soft tissue–equivalent space-occupying mass of the left maxillary region was identified with an extent of 2.4 × 2.8 × 1.5 cc and extension into the maxillary sinus. The OPG showed osteolytic regions of the left maxilla and right mandibular side. Complementary CBCT showed extensive osteolysis of the left maxillary alveolar process in the molar region. Several teeth were within the osteolytic zone. The tooth roots were intact on the slice images. In addition, a conspicuous osseous lesion was recorded in the right mandibular angle. This second osteolytic finding was without contact with the dentition. Figures 1 and 2 illustrate relevant radiological findings.

### Therapy

**Fig. 3:**
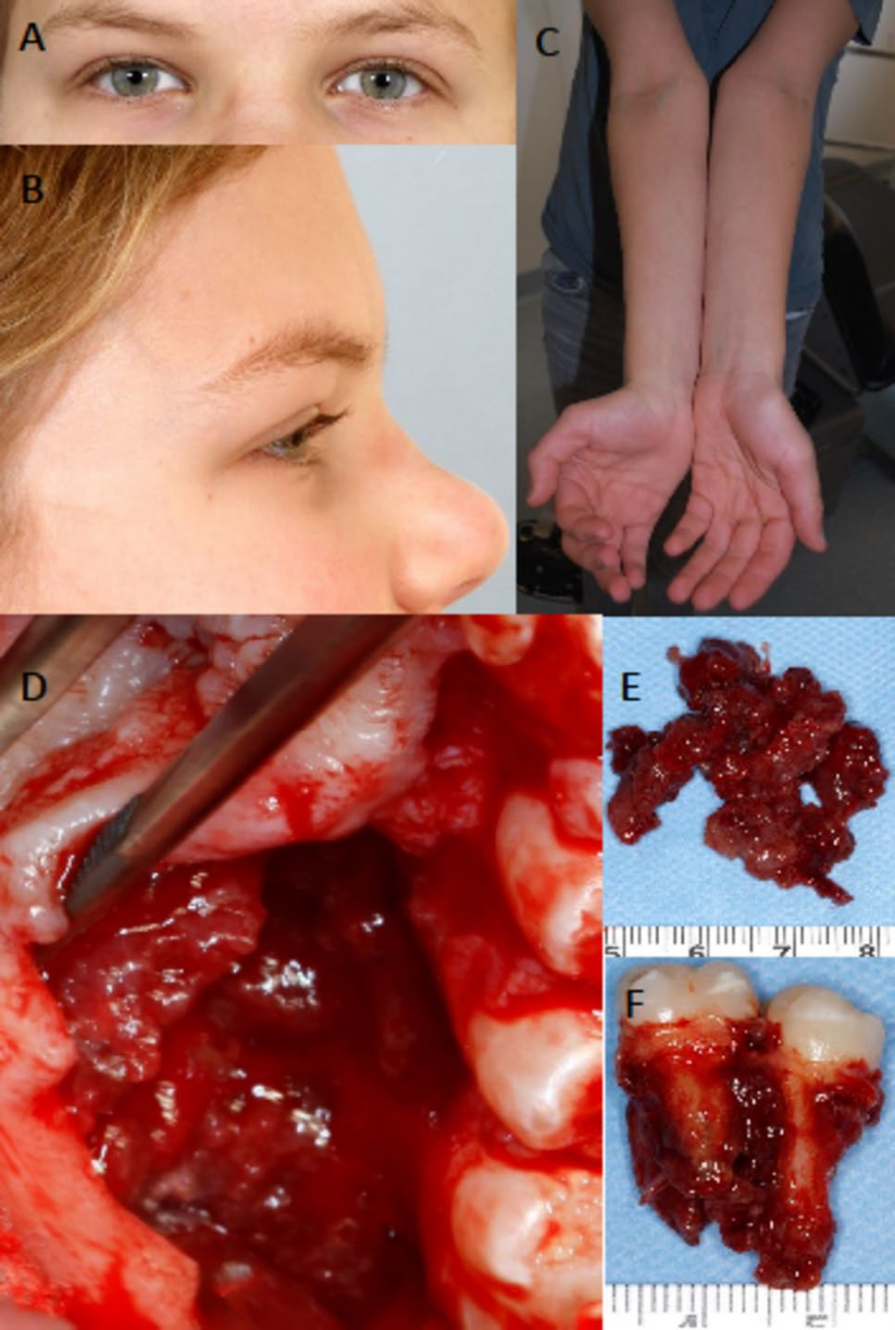
Physical findings (**A**–**C**) and surgical exploration (**D**–**F**) in a Noonan syndrome patient with central giant cell granuloma of the jaws. (**A**) Increased intercanthal distance feigns hypertelorism of the orbits. (**B**) Prominent forehead and retracted transition from forehead to nasal bridge are common findings in RASopathies. (**C**) Cubitus valgus. (**D**) Under the elevated palatal mucosa, the lesion of the maxilla is interspersed with fragmentary bone remnants (**E**) surrounding both fully developed, intact molars (**F**)

Clinical examination was insufficient for diagnosis. Localization and findings could alternatively be signs of an osteolytic neoplasm as well as a primary osteogenic lesion. However, the finding of a synchronous second mandibular lesion on CBCT was suggestive of a syndromic cause. A biopsy of the connective tissue lesion brought the diagnosis of CGCG. Subsequently, under general anesthesia, the lesion of the maxilla including teeth 25 and 26 was resected (Fig. 3). The lesion of the right mandibular angle was osteotomized, the soft tissue tumor scraped out, and the uppermost bone layer of the lesion was surgically removed. At both sites, the bone was replaced by soft, granular, slightly bleeding tumor tissue. Soft tissue wound healing was unremarkable and *per primam*. In the radiological follow-up, a maxillary tumor recurrence was suspected 6 weeks after surgery. Revision of the situs and excision of the soft tissue lesion confirmed the diagnosis of recurrent maxillary CGCG. No signs of local tumor growth have been observed for 30 months follow-up following the second intervention (Fig. 2). Consequently, the patient’s orthodontic treatment was initiated to reduce the interdental space. Figure 3 illustrates relevant clinical findings.

Substitution with Decristol™ normalized the serum value of vitamin D3 within 6 months. Initially, it was suspected that the patient’s disease was a RASopathy caused by a *RAS* mutation. However, no mutations were detected in *HRAS*, *KRAS*, and *NRAS*. Further molecular genetic examination revealed a heterozygous missense variant c.767A > G [p.(Gln256Arg)] on exon 7 of *PTPN11* and exclusion of further mutations in *BRAF*, *CBL*, *MAP2K1/MAP2K2*, *RAF1*, *SHOC*2, and *SOS1*. The *PTPN11* mutation has previously been described as pathogenic and causative of NS. There are no known diseases in parents or other relatives that could indicate NS. Pediatric cardiac examination revealed normal findings consistent with age.

### Follow-up

Up to now, no maxillary local recurrence occurred. So far, re-ossification of the lesion in the right mandibular angle is complete in the anterior ramus region but incomplete in the region surrounding the mandibular canal inferior to the mandibular foramen. A residual lesion cannot be excluded with certainty. Regular clinical and radiological examinations (first year: quarterly, then semi-annually) have demonstrated the stability of the surgical outcome. The development of the root of the left upper wisdom tooth and the mesial migration of the tooth into the spontaneously formed new jawbone in the resection region of the two extracted molars is impressive (Fig. 2).

The patient’s height has increased by 10 cm during the last 2 years. The widened intercanthal distance persists.

## Discussion

The report describes the rare and diagnostically relevant jaw findings in a case of so far unrecognized NS. The diverse symptoms of NS can delay the diagnosis in individual cases because initial diagnosis and therapy of presumed unrelated findings are the focus of medical activity. The case presented here is unusual because only in rare cases the diagnosis of CGCG precedes the identification of the syndrome or the oral evaluation set the diagnostic pathway in motion [9]. The report indicates that a review of oral findings can help diagnose the syndrome. Following syndrome diagnosis, patient care can assess typical disease-associated risks and provide medical prophylaxis.

NS is considered a disease caused by monogenic mutation. However, mutations in several genes have been identified causing the phenotype of this syndrome. In most NS patients, autosomal-dominant inheritance patterns are identified. A *PTPN11* mutation is detected in approximately 50% of NS patients. Mutations in other genes are significantly rarer [1, 10]. The mutation identified here has already been detected in NS and is considered disease-causing. However, it is currently unknown which local cellular alterations lead to the development of benign osseous neoplasms in the presence of a pathogenic germline mutation. This reference is relevant because in another RASopathy with the occasional occurrence of CGCG (neurofibromatosis type 1), bi-allelic loss of function of the causal gene obviously is necessary for the development of the jaw lesion [6, 11].

Great attention is paid to the skull and facial findings in the diagnosis of NS, such as trigonocephaly, and light-colored and widely spaced eyes [1]. Hypertelorism is considered a constant clinical feature with a prevalence of approximately more than 80% of affected individuals. However, in the descriptions of NS-associated increased interorbital ratios, it often remains unclear whether the term “hypertelorism” means an increased intercanthal distance or a measurable increase in the distance between the medial orbital walls defining *orbital* hypertelorism [12]. In automatic face recognition software for the identification of NS patients, measurement points set in the periorbital area refer to the intercanthal distance [13]. It is plausible to assume that the increased eye distance in most publications on the subject refers to the impression of eyes set unusually wide apart as defined by the more distant position of the two inner lid angles: many NS patients develop epicanthic folds [14]. The increased intercanthal distance in the presented case can be explained by the sagittal underdeveloped nasal bridge and prominent forehead. Measured interorbital distance indicates a normal value [15, 16].

### CGCG phenotype

In contrast to NS, CGCG is rarely diagnosed. A population genetic study has estimated the prevalence of CGCG to be 1:1,000,000 [17]. Information on the prevalence of CGCG in RASopathies is not yet available. In RASopathies, the detection of CGCG is probably also a rare finding [11] and the frequency likely depends on the specialization of the reporting institution. Thus, the reported prevalence of 10% CGCG in NS patients in the collected statistics of one clinic is probably influenced by the institution’s specialization in jaw lesions treatment [12] and not a reliable source for assessing the syndrome-associated frequency of the lesion.

In general, the mandible is far more frequently affected by CGCG than the maxilla [8]. Extragnathic CGCG has been reported, but without information on the genetic status of the patients or indications of somatic mutations of the lesions [18].

Non-aggressive CGCG variants appear to be characterized by the absence of tooth resorption [19]. In a meta-analysis of CGCG reviews of non-syndromic cases, tooth resorption was reported to be 22.8% [8]. Palatal swelling and tooth loosening were each recorded in 2 of 26 cases in a retrospective CGCG study [19]. A systematic survey of sporadic CGCG is not informative of the frequency of this important clinical finding [8].

In a recent meta-analysis on the development of CGCG, it was assumed that the pathogenesis of the lesions differs between sporadic and syndrome-associated cases. The study was on 2270 reported cases in the literature [8]. Multiple CGCG was evaluated as an indication of a syndromic disease and excluded from further study. Indeed, van den Berg et al. emphasized that multifocal CGCG almost only occurs in syndromes [20, 21]. However, unilocular lesion does not exclude a syndromic background of bone disease [22]. Therefore, (unrecognized) syndromic cases were probably included in the meta-analysis [8]. However, the resulting exclusion of syndromic CGCG in the literature analysis [8] makes the findings less meaningful for the clinical assessment of syndromic cases. On the other hand, in presumably syndromic cases of multiple CGCT of the jaws, the genetic detection of a mutation typical for these diseases (cherubism, Noonan) is not successful in all cases [23]. Clinical clues for non-syndromic aggressive lesions were cortical bone perforation and tooth root resorption. Evidence of tooth resorption and tooth displacement was the only indication of an increased risk of recurrence comparing aggressive and non-aggressive lesions [24]. The size of the lesion did not affect the risk of recurrence [24].

Treatment of CGCG is surgical [12]. However, local recurrences after ablative therapy are well known and had to be treated in the maxillary region in this case as well. Alternative pharmacological therapies have been tried but have not yet achieved general acceptance.

Non-surgical Treatment. A recent systematic review analyzed the success of non-surgical treatment of CGCG. The review of 15 studies on this topic led the authors to conclude that various drugs could be successfully used to reduce lesion size in at least some patients and that re-ossification had been demonstrated in some cases. However, the authors do not address whether the drug effects were influenced by the genetic status of the patients [25].

A recent prospective clinical study presented the treatment results of eight CGCG patients treated with denosumab (single dose: 120 mg) [26]. Ossification of lesion was observed in all cases, in three cases combined with volume reduction. However, 12 months after completion of therapy, recurrence was observed in four of seven patients with complete drug therapy. Apparently, three patients had maxillofacial surgical intervention after drug treatment. The authors point out that toxic effects under denosumab on electrolyte metabolism require continuous medical monitoring. Denosumab may cause hypocalcemia, especially in patients with vitamin D deficiency. The study does not indicate whether syndromic cases of CGCG were treated in the patient population [26]. In another case report, denosumab was successfully used in two sisters with multilocular CGCG. The course was clinically documented over 4 years, during which neither recurrence nor osteonecrosis occurred. Genetic testing showed no mutation of R234X, exon 7 of the HRPT2 gene [23].

Experience with four NS patients of growth age with multiple CGCG under denosumab therapy describes clinical and radiological response to the medication over an application period of maximum 2 years. Adverse drug reactions at therapy initiation were hypocalcemia and joint pain. After discontinuation of treatment, a rebound hypercalcemia was frequently noted. Noteworthy, all 4 patients were additionally treated with bisphosphonates after cessation of denosumab for the treatment of both hypercalcaemia and increased bone resorption [27]. In one of the four cases, treatment with growth hormone was continued with denosumab [27}. The order of drugs (bisphosphonate(s) and denosumab) administered sequentially for osteoporosis treatment appears to influence the risk of drug-associated osteonecrosis [28]. Regarding the aforementioned casuistic contributions to the denosumab therapy of CGCG in both sporadic and syndromic CGCG, the question remains unresolved as to whether this drug therapy represents an effective alternative to surgical therapy with few side effects.

Drug treatment of bone lesions with osteoclast-inhibiting drugs has proven to be particularly effective in the oncological treatment of bone metastases from malignant diseases. A further and increasing indication for this functionally defined group of drugs is the treatment of osteoporosis. Osteonecrosis of the jaw is a rare, known adverse drug reaction (ADR) of osteoclast-inhibiting drugs, particularly bisphosphonates and denosumab. In recent years, denosumab-associated osteonecrosis of the jaw has taken the lead among these jaw-specific ADRs [29]. The use of denosumab remains an off-label use for patients with sporadic [30] and Noonan-associated [31] CGCG. Denosumab-associated derailment of calcium metabolism can have serious health consequences [27–31]. Currently, data are weak to recommend the use of osteoclast-inhibiting drugs in syndromic CGCG, especially when other endocrine abnormalities affecting bone metabolism have to be considered.

Bone health of NS patients has been poorly studied [32]. Vitamin D deficiency or insufficiency is observed in approximately half of Noonan children [33]. As the case presented shows, bone formation, especially of young patents after sufficient surgical excavation of the lesions, is impressive, but requires regular monitoring of the local findings. The presented case report documents the rapid correction of vitamin D concentration after oral substitution and a significant increase in body size during the observation interval.

## Conclusion

CGCG is a rare disease of the jaws. Recent studies show that the lesions are caused by mutations in genes involved in controlling the *RAS* signaling pathway. CGCG occurs both sporadically and as syndromic lesions. The treatment of lesions in patients, who are often adolescents, can have a significant impact on dental and osseous functions. Surgical treatment, especially of smaller lesions, is preferable. In the case of larger lesions, an assessment of the loss of function and esthetic consequences and adverse reactions of drugs are important factors in the treatment decision [34]. Detection of CGCG of the jaws in an individual should prompt further investigation.
